# Utilising NV based quantum sensing for velocimetry at the nanoscale

**DOI:** 10.1038/s41598-020-61095-y

**Published:** 2020-03-24

**Authors:** Daniel Cohen, Ramil Nigmatullin, Oded Kenneth, Fedor Jelezko, Maxim Khodas, Alex Retzker

**Affiliations:** 1 Racah Institute of Physics, The Hebrew University of Jerusalem, Jerusalem, 91904 Givat Ram, Israel; 2 Complex Systems Research Group, Faculty of Engineering and IT, The University of Sydney, Sydney, New South Wales 2006 Australia; 3 Dept. of Physics, Technion, Israel; 4 Institute for Quantum Optics, Ulm University, Albert-Einstein-Allee 11, 89081 Ulm, Germany; 5 Center for Engineered Quantum Systems, Dept. of Physics & Astronomy, Macquarie University, 2109 NSW, Australia

**Keywords:** Quantum metrology, Theoretical physics, Nanofluidics

## Abstract

Nitrogen-Vacancy (NV) centers in diamonds have been shown in recent years to be excellent magnetometers on the nanoscale. One of the recent applications of the quantum sensor is retrieving the Nuclear Magnetic Resonance (NMR) spectrum of a minute sample, whose net polarization is well below the Signal-to-Noise Ratio (SNR) of classic devices. The information in the magnetic noise of diffusing particles has also been shown in decoherence spectroscopy approaches to provide a method for measuring different physical parameters. Similar noise is induced on the NV center by a flowing liquid. However, when the noise created by diffusion effects is more dominant than the noise of the drift, it is unclear whether the velocity can be efficiently estimated. Here we propose a non-intrusive setup for measuring the drift velocity near the surface of a flow channel based on magnetic field quantum sensing using NV centers. We provide a detailed analysis of the sensitivity for different measurement protocols, and we show that our nanoscale velocimetry scheme outperforms current fluorescence based approaches even when diffusion noise is dominant. Our scheme can be applied for the investigation of microfluidic channels, where the drift velocity is usually low and the flow properties are currently unclear. A better understanding of these properties is essential for the future development of microfluidic and nanofluidic infrastructures.

## Introduction

The interest in NV centers has rapidly increased in the past decade. This is because this point defect in a diamond can be used as a quantum register with simple optical initialization and readout even at room temperature^1^. The NV center has been used for many applications, such as quantum computation^2–4^, hyper-polarization^5–9^ and magnetic field sensing^10–14^. The latter has evolved recently into nanoscale Nuclear Magnetic Resonance (NMR) spectroscopy of small samples with polarization well below the detectable Signal-to-Noise Ratio (SNR) of classic NMR devices^15–21^. The NV’s ability to sense minute magnetic fields makes it prone to decoherence by fluctuating fields. The decoherence time is dependent on the sample’s properties and can then be used to estimate, for example, the NV’s depth^22^, the liquid’s diffusion coefficient^23^ and ion concentrations^24^. When considering an NV center in proximity to a drifting liquid, a similar fluctuating field is induced on the NV. Its decoherence time will then depend on the drift velocity. Here, we propose and analyze this measurement technique for the mean drift velocity and the self-diffusion coefficient inside a flow channel based on NV center NMR spectroscopy. This technique is of special importance to the field of microfluidics.

Microfluidics are now a well-established platform, that has revolutionized bio-medical research, primarily as a result of the Lab-on-a-Chip approach to chemical and biological analysis^25–32^. Microfludic channels present rich physical phenomena which depend on a large number of parameters (geometry, fluid type, fabricated material, etc.). Understanding the physics of these channels is key to the future development of the field, not only for the fabrication of such devices, but also because their research applications demand both high temporal and spatial measurement resolution, which require an in-depth understanding of their flow characteristics^32,33^. One of the main areas of interest is the flow profile near the boundary of microfludic channels, since these boundary effects play a major role when attempting to downsize these structures to the nanoscale regime. In recent years accumulating evidence has shown that the flow in such channels does not always obey the no-slip boundary condition^33–35^, which is commonly used to solve the Navier-Stokes equations. Though there have been advances in the microscopic theory of this phenomenon^35^, an experimental method to accurately measure the velocity near the surface has not yet been developed. Our proposed setup is sensitive to surface effects and therefore meets this need.

**Figure 1:**
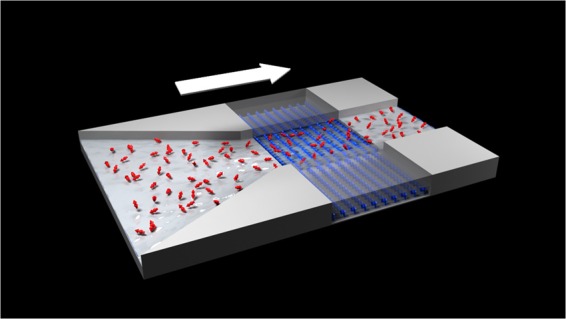
*The scheme*– The nuclear spins (red arrows), which are randomly oriented, generate a random magnetic field on an ensemble of NV centers (blue arrows), which are located near the surface of the diamond. The NVs sense the magnetic field, whose randomness is amplified by the flow. By optically measuring the state of the NV centers, the power spectrum of the magnetic noise can be estimated, from which the velocity can be deduced. Created by Studio Mirage, Israel.

The main idea is sketched in Fig. 1, where a flow through a microfluidic channel is sensed by an NV center ensemble, in a setup similar to the one that was used in prior works^23,24^. The unpolarized spins motion induces a random magnetic field at the location of the NV centers. The power spectrum of the magnetic field noise can be estimated by optically probing the NVs. By analyzing the noise characteristics, the flow properties can be deduced. It should be noted that velocimetry methods using classic NMR techniques exist^36–38^. In principle, these methods can also be applied in the nanoscale NMR settings. The implementation of this type of experiment is challenging, since it requires efficient polarization of the nuclear spins together with a strong, stable magnetic field gradient.

In this work, we show that a nanoscale NMR approach for velocity estimation in microfludic channels is possible without polarization and even when diffusion dominates the dynamics. The remainder of this paper is organized as follows. First, we discuss measurement protocols. Then, we estimate the sensitivity for the velocity measurement assuming a Lorentzian noise spectrum. We proceed by showing that in fact the spectrum deviates from the Lorentzian approximation due to the diffusion process and that the corrected spectrum leads to improved sensitivity even when the diffusion process is more dominant than the drift. Finally, we present molecular dynamics simulations that support our analytic results and compare our method to current velocimety techniques.

## Results

### Measurement scheme

**Figure 2:**
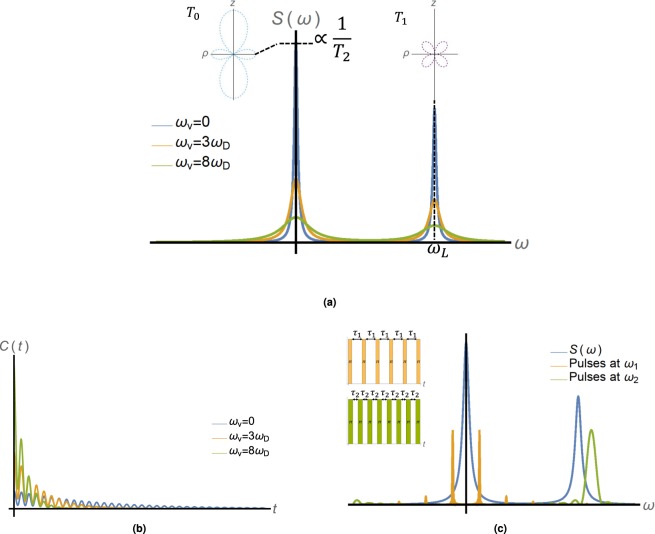
(**a**) The Lorentzian power spectrum at different velocities - The power spectrum consists of multiple peaks associated with different parts of the dipole-dipole interaction between the NV and the ensemble. Shown here are the zero frequency term, *T*_0_ ∝ *S*_*z*_*I*_*z*_ term (dotted light blue), and the Larmor frequency terms *T*_±1_ ∝ *S*_*z*_*I*_±_ (dotted purple). Each point on the spectrum is associated with a different decay rate of the NV’s quantum state; for example the zero frequency peak is proportional to the NV’s pure dephasing time *T*_2_. The velocity changes the width and the peak of the spectrum, and therefore it can be deduced by measuring them. (**b**) The correlation function at different velocities - the velocity can also be estimated by directly measuring the correlation function, the Fourier transform of the power spectrum. The correlation oscillates at the Larmor frequency, and decays in a rate that depends on the velocity and diffusion. The peak of the correlation is proportional to ${\tau }_{c}{\gamma }_{e}^{2}{B}_{RMS}^{2}$, where *τ*_*c*_ is the correlation time, which is also determined by the velocity. (**c**) While the spectrum (blue) at zero frequency can be measured in a Hahn-echo experiment, different parts of the spectrum require external driving of the NV. The Fourier transform of the pulse sequence acts as a window function on the the power spectrum, such that the dephasing time *T*_2_ is given by a convolution between the window function and the power spectrum. Shown above are two examples of window functions at frequencies ${\omega }_{1/2}=\frac{\pi }{{\tau }_{1/2}}$ (orange/green) that sample different parts of the spectrum. The window functions peak sharply at the characteristic frequency of the external drive and they have smaller peaks at higher harmonics of that frequency. Both the window functions and the power spectrum are symmetric with respect to *ω* = 0.

We consider the NV center as a two level system (spin $\frac{1}{2}$) with an energy gap of *γ*_*e*_*B*_*ext*_, where *B*_*ext*_ is the externally applied magnetic field^1^. The power spectrum generated by the motion can be read by a decoherence spectroscopy method^39–44^ or spin relaxation^45,46^. These methods exploit the fact that the transition rate or relaxation time in a two-level system is proportional to the spectral density evaluated at the transition frequency or the Rabi frequency of an external drive. In both cases, the relaxation rate is given by Γ = *S*(*ω*), where *S*(*ω*) is the power spectrum at frequency *ω*, which is either the external drive’s Rabi frequency or the energy gap of the two level system (see Fig. 2). The relaxation rate and, therefore, the power spectrum can be efficiently estimated from the population decay. Since all the effects of the velocity emerge from the magnetic noise induced on the NV, polarization is not essential here and the velocity can be measured in unpolarized samples in a way similar to diffusion measurements^22^.

The different components of the magnetic field can be sensed by probing the different parts of the dipole-dipole interaction separately. We denote *S*_*i *_(*I*_*i*_) as the spin operator of the NV (nuclear spin) at the *i* direction, *S*_±_(*I*_±_) as the NV’s (nuclear spin’s) raising/lowering operators and *θ*_*i*_ as the angle between the NV’s magnetization axis to the vector connecting the NV to the *i*-th nucleus. The velocity can be sensed by the ${T}_{0}=(3\cos {(\theta )}^{2}-1){S}_{z}{I}_{z}$ term or the ${T}_{\pm 1}=\frac{3}{2}\sin \theta \cos \theta {e}^{\mp i\phi }\left({S}_{z}{I}_{\pm }+{S}_{\pm }{I}_{z}\right)$ (see for example^47^) as shown in Fig. 2. The *T*_0_ is the classical term derived from the thermal polarization fluctuations of nuclei within a volume of *d*^3^, which inflict a random field on the NV. In the case of a polarized fluid, the noise is decreased and this term vanishes. However, probing the dynamics is still possible if the nuclei are polarized in the *x* − *y* plane. This is demonstrated in the Supplementary Information (SI).

The second term, *T*_±1_, is a rotating term which manifests itself at high frequencies and is routinely used to probe the nano-NMR spectrum^16,18,20,21^. In order to probe this term, a rotation of the NV at the Larmor frequency must be introduced, for example, by pulsed dynamical decoupling^5,15–19,48^, which yields the following effective Hamiltonian: ${H}_{1}=\frac{3}{2}\sin \theta \ \cos \theta {S}_{z}\left({I}_{x}\cos (\delta t+\phi )+{I}_{y}\sin (\delta t+\phi )\right),$ where *δ* is the frequency difference between the Larmor frequency and the frequency of the pulses. The velocity can be estimated also by measuring the correlation function of the magnetic field directly. The idea is presented in Fig. 2b and discussed further later on.

### Lorentzian model

The sensitivity of the velocity estimation can be understood in a simplified model. In this nano-NMR setting there are two characteristic time scales: the diffusion time scale ${\tau }_{D}=\frac{{d}^{2}}{D}$ and the drift time scale ${\tau }_{v}=\frac{d}{v}$, where *d* is the NV’s depth below the diamond surface, *D* is the self-diffusion coefficient and *v* is the mean drift velocity. These time scales control the correlation time of the NMR signal if only one of the processes is dominant. Respectively, they are the inverse characteristic widths of the NMR spectra. In the case where both processes are significant, the natural expectation is that the characteristic width is ${\sigma }^{2}={({\tau }_{D}^{-1})}^{2}+{({\tau }_{v}^{-1})}^{2}\equiv {\omega }_{D}^{2}+{\omega }_{v}^{2}.$ In order to estimate the sensitivity we follow the commonly accepted assumption that the nano-NMR spectrum is a Lorentzian function^20,22^, $S(\omega )=\frac{{\sigma }^{2}S(\omega =0)}{{\omega }^{2}+{\sigma }^{2}}$. The peak is given by $S(\omega =0)={\gamma }_{e}^{2}\frac{{B}_{RMS}^{2}}{\sigma }$, and ${B}_{RMS}^{2}=n{\left(\frac{{\mu }_{0}\hslash {\gamma }_{N}}{4\pi }\right)}^{2}\frac{1}{{d}^{3}}$, where *γ*_*e*∕*N*_ is the electron/nuclear gyromagnetic ratio, *μ*_0_ is the vacuum permeability, *ℏ* is the reduced Plank constant and *n* is the nuclei number density. A 5 nm deep NV and water density of 33 nm^−3^ result in *B*_*RMS*_ ≈ 250kHz∕*γ*_*e*_. The sensitivity is best described by the dimensionless quantities:

1 $$\mathop{S}\limits^{ \sim }(\omega )=\frac{S(\omega )}{{\gamma }_{e}^{2}{B}_{RMS}^{2}{\tau }_{D}},\,\mathop{v}\limits^{ \sim }=\frac{v}{d/{\tau }_{D}}.$$

The first denominator can be interpreted as a unit power spectrum taken as *S*(*ω* = 0, *v* = 0), whereas the second denominator is a unit velocity for which *ω*_*v*_ = *ω*_*D*_, i.e., ${\tilde{v}}=\frac{{\omega }_{v}}{{\omega }_{D}}$. We use these units henceforth in our estimations.

The decay rate Γ of the NV can be estimated with a sensitivity of ~Γ for a single shot measurement within one experiment which lasts for *t* ~ Γ^−1^. Repeating this measurement for a total duration of *T* results in a sensitivity of $\Delta \Gamma =\sqrt{\frac{\Gamma }{T}}$. Since most NV measurements are not single shot, the sensitivity is reduced by an order of magnitude compared to this estimate. The standard regime is the one in which the velocity effect is small compared to the diffusion effect; i.e., *ω*_*v*_ ≪ *ω*_*D*_, therefore the decay rate equals $\widetilde{S}\approx 1-\frac{{{\tilde{v}}}^{2}}{2},$ which is quadratic in the velocity; hence, the sensitivity with respect to the velocity is substantially reduced in this regime. This leads to the natural conclusion that whenever the effect of diffusion is larger than the velocity effect, it is very challenging to estimate the velocity. This can be intuitively understood in the following way. The velocity is estimated from changes in the magnetic field due to the drift of the fluid, as in Fig. 1. Diffusion, however, causes the magnetic field to change as well. When the molecules move away from the vicinity of the NV due to diffusion rather than drift, the effect of the drift is suppressed.

Before we proceed, let us quantify this effect. The sensitivity of the velocity estimation is optimal at *ω* = 0. Therefore, the uncertainty in the velocity,  $\frac{\sqrt{S\left(\omega =0\right)}}{\sqrt{T}}\frac{1}{\left|\frac{\partial S(\omega =0)}{\partial v}\right|}$, assuming *ω*_*v*_ ≪ *ω*_*D*_, is

2 $$\Delta \mathop{v}\limits^{ \sim }=\frac{1}{\mathop{v}\limits^{ \sim }}\frac{1}{{\gamma }_{e}{B}_{RMS}{\tau }_{D}^{1/2}\sqrt{T}}=\frac{1}{\mathop{v}\limits^{ \sim }}\frac{1}{\sqrt{S(\omega =0,v=0)T}}.$$

This can also be rewritten as $\Delta {\tilde{v}}=\frac{1}{{\tilde{v}}\phi \sqrt{M}},$ where *ϕ* is the phase collected during a time *τ*_*D*_ and *M* is the number of measurements. For the experimental parameters of water: *D* ~ 2.3 ⋅ 10^3^nm^2^ *μ*s^−1^, *n* ~ 33nm^−3^ ^49^ and *d* ~ 10nm, *v* = 1mm s^−1^, the sensitivity is approximately: $\frac{\Delta v}{v}\approx 3\frac{1{0}^{3}\,{{\rm{s}}}^{1/2}}{\sqrt{T}}$. Limited contrast and collection efficiency will reduce the sensitivity by a factor of 10, therefore the sensitivity achieved by a single NV, henceforth denoted by a subscript *s*, is $\frac{\Delta {v}_{s}}{v}\approx 3\frac{1{0}^{4}\ {{\rm{s}}}^{1/2}}{\sqrt{T}}$. This can be mitigated by collection from many NVs, which increases the sensitivity by a factor of $100\sqrt{\frac{A}{\mu {{\rm{m}}}^{2}}}$, where *A* is the area covered by the NV centers. Thus, in total $\frac{\Delta {v}_{ens}}{v}\approx 300\,\frac{{{\rm{s}}}^{1/2}}{\sqrt{T}}\sqrt{\frac{{(\mu {\rm{m}})}^{2}}{A}},$ which requires a large NV area $\sim {\left(150\mu {\rm{m}}\right)}^{2}$ to get reasonable sensitivity. Note that we assume an NV density of 10^12^ cm^−2^, which is about the current upper bound for shallow NV ensembles^50^. Therefore it should be interpreted as an upper limit. Differences in the distance from the surface in the NV ensemble result in negligible changes in sensitivity (see SI).

In the following we show that the Lorentzian approximation of the power spectrum is very crude since in fact the power spectrum is linear or proportional to the square root of the velocity, depending on the frequency regime (see Methods). This enhances the sensitivity (Eq. 2) by a factor of up to ${\tilde{v}}^{3/2}$ (see Eq. 5 in sec. 2.4). For water flowing in a microfluidic channel ${\tilde{v}}\approx 5\cdot 1{0}^{-3}$, which implies a decrease in the uncertainty by a factor of ${\tilde{v}}^{3/2}\approx 3\cdot 1{0}^{-4}$ compared to eq. 2.

**Figure 3:**
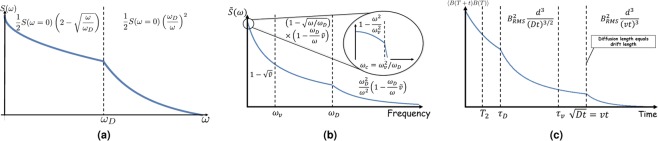
(**a**) The power spectrum for diffusion only. The power spectrum is divided into two regimes which are defined by *ω*_*D*_. The regime *ω* ≫ *ω*_*D*_ is characterized by the expected Lorenzian type decay. The *ω* ≪ *ω*_*D*_ regime is described by a $\sqrt{\omega }$ decay. (**b**) The structure of the power spectrum for diffusion and drift in normalized units. The power spectrum has three different regimes which are separated by *ω*_*v*_ and *ω*_*D*_. The high frequency part *ω* > *ω*_*D*_ is roughly equal to the power spectrum at zero velocity and damped by a factor of about $\left(1-{\tilde{v}}\right)$. This also holds for the intermediate region, *ω*_*v*_ < *ω* < *ω*_*D*_. The low frequency part is nearly a constant, which equals ${S}_{v=0}\left(\omega ={\omega }_{v}\right)$. Near zero the power spectrum decays quadratically as expected by the behavior of the correlation function. (**c**) The structure of the correlation function. The decay of the correlations changes at the point where the diffusion distance equals the drift distance, $t=\frac{D}{{v}^{2}}$. In the case where the correlation does not decay substantially within an NV coherence time, *T*_2_, the correlations in the NV measurements are proportional to the magnetic correlations.

A heuristic sketch of the power spectrum without drift is shown in Fig. 3. While a Lorentzian type behavior is valid for large frequencies, the behavior for low frequencies deviates considerably from a Lorentzian and decays as $1-\sqrt{\omega /{\omega }_{D}}.$ This behavior derives from the fact that the correlation function at long times decays as *t*^−3∕2^ due to diffusion dynamics which transforms to $\sqrt{\omega }$ dependence in the power spectrum. This non-Lorentzian behavior has been observed experimentally for diffusing Rb vapor in N_2_ gas^51,52^. In the following we show that this non-analytic behavior has important implications for parameter estimation.

The velocity can be estimated either from the correlation function or from the power spectrum. Although these two quantities are related via the Fourier transform, the sensitivity obtained from each method is different, because of the modifications in the experimental procedure for probing them. The optimal strategy corresponds to different regimes of the coherence time of the NV center, *T*_2_. In the case where the coherence time of the NV center is shorter than the correlation time of the signal, it is advantageous to probe the correlation function, since in this regime each measurement probes approximately a constant magnetic field. Therefore, the correlation between measurement results is proportional to the magnetic correlation function. In the opposite regime it is best to probe the power spectrum directly.

The coherence time is dictated by external noise factors, such as magnetic impurities, that are not taken into account in our estimation. In shallow NVs the main source of noise is due to surface effects^53–56^, while the dynamics is controlled by magnetic impurities for bulk NVs^57–59^. Though the coherence time of single NVs has gradually increased in past years, due to surface engineering, annealing techniques and sophisticated pulse sequences, the task remains challenging for a dense ensemble^50^. This will not effect the estimation via the correlation function, as it assumes that *T*_2_ is the smallest time scale of the system. It can, however, impact the power spectrum estimation, as it requires *T*_2_ to be larger than *τ*_*D*_. Hence, when performing an experiment, an optimization will be required to achieve the best sensitivity.

We henceforth assume a reasonable NV coherence time is 100 *μ*s^20^ after dynamical decoupling. Since achieving such coherence times might suggest taking lower NV density, the numeric estimations via the power spectrum might deviate by an order of magnitude.

### Estimation via the correlation function

The general structure of the correlation function, based on secs. V and VI of the SI and the Methods section, is shown in Fig. 3c. In the case where *τ*_*D*_ is longer than the coherence time of the NV, which is valid for viscous fluids such as oil and NVs deeper than 5 nm, each measurement result is a function of the instantaneous magnetic field, thus making it advantageous to probe the correlation function. The time separation that yields information about the velocity can be found in the regime in which the drift length is larger than the diffusion length ($vt > \sqrt{Dt}$). In this regime, the phase acquired by the NV during one measurement is *ϕ* ≈ *γ*_*e*_*B*(*t*)*T*_2_, and thus the correlation function is $\langle {P}_{\uparrow }({t}_{1}){P}_{\uparrow }({t}_{1}+t)\rangle \approx \langle \frac{1+\sin \phi ({t}_{1})}{2}\frac{1+\sin \phi ({t}_{1}+t)}{2}\rangle \approx \frac{1}{2}+\frac{1}{2}\langle {\phi }_{\uparrow }({t}_{1}){\phi }_{\uparrow }({t}_{1}+t)\rangle \approx \frac{1}{2}+\frac{1}{2}{T}_{2}^{2}{\gamma }_{e}^{2}{B}_{RMS}^{2}\frac{{d}^{3}}{{(vt)}^{3}}.$ Assuming that *vt* ≈ *d* the uncertainty is $\frac{\Delta v}{v}=\frac{1}{3}\frac{1}{{T}_{2}^{2}{\gamma }_{e}^{2}{B}_{RMS}^{2}}.$ As the velocity in oil can reach 10^−2^ mms^−1^, yielding an optimal depth of 25 nm and taking into account a limited collection efficiency, a fractional uncertainty of $\frac{\Delta {v}_{s}}{v}=5\cdot 1{0}^{-2}$ is achieved. Thus, the total uncertainty is on the order of $\frac{\Delta {v}_{ens}}{v}=5\cdot 1{0}^{-3}\frac{\sqrt{{\rm{s}}}}{\sqrt{T}}\sqrt{\frac{{(\mu {\rm{m}})}^{2}}{A}}.$ The limitation is that this method only works as long as *T*_2_ is shorter than *τ*_*D*_ and *τ*_*v*_, which only applies to very viscous fluids. For example, for an oil and NV depth of 25 nm, the diffusion time *τ*_*D*_ ≈ ms^6^. For water on the other hand, and for a shallower NV, *τ*_*D*_ = 0.02 *μ*s, which reduces the sensitivity by four orders of magnitude.

### Estimation via the power spectrum

**Figure 4:**
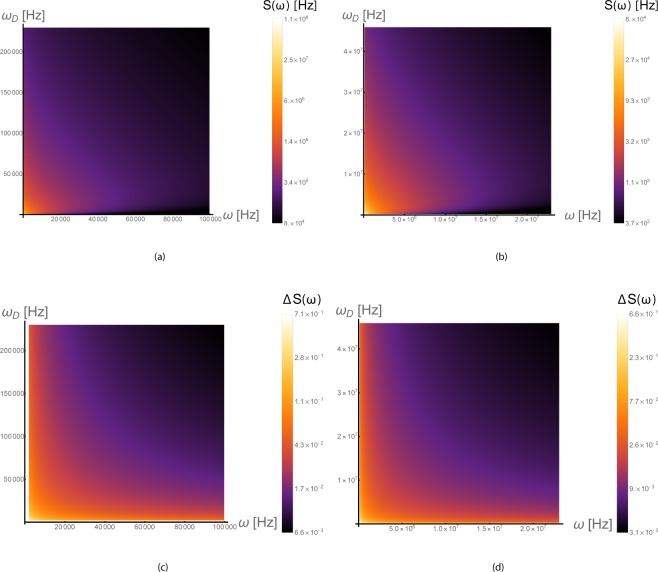
(**a,b**) The power spectrum of freely diffusing particles (without drift) as a function of the frequency and the diffusion frequency at *d* = 10nm given by Eq. 15. The lower bound of the diffusion frequency at (a/b) fits a diffusion coefficient of 0.1*D*_*oil*_/0.01*D*_*water*_ and the upper bound fits 0.01*D*_*water*_/2*D*_*water*_. At zero frequency the power spectrum decays as a function of *ω*_*D*_ because it is proportional to the correlation time *τ*_*D*_. At other frequencies the spectrum first increases with *ω*_*D*_ when the Lorentzian behavior is dominant, and then decreases again when *ω* ≪ *ω*_*D*_. (**c,d**) The normalized contrast $\Delta S(\omega )=1-\frac{{S}_{v}(\omega )}{{S}_{v=0}(\omega )}$ with finite drift velocity as a function of the frequency and the diffusion frequency at *d* = 10 nm. The power spectrum is given by Eq. 23 for *ω* ≤ *ω*_*v*_ and by Eq. 22 for *ω*_*v*_ < *ω* with *C* = 1. The bounds of (*c*/*d*) match those of (*a*/*b*) and the drift velocity is taken to be $0.01/1\,\frac{{\rm{mm}}}{{\rm{s}}}$. The contrast decays gradually as a function of the two frequencies. The strong contrast results in an improved sensitivity scaling.

For lower viscosity fluids it is advantageous to estimate the velocity directly from the power spectrum. The structure of the power spectrum, based on secs. II and IX of the SI and the Methods section, is shown in Fig. 3b and its behavior is divided into three regimes: the low frequency regime *ω* < *ω*_*v*_, the intermediate regime *ω*_*v*_ < *ω* < *ω*_*D*_ and the high frequency regime *ω* > *ω*_*D*_. In the high and intermediate frequency regimes the power spectrum is linear in velocity and in the low frequency regime it has a square root dependence. Further exploration of the parameter space is shown if Fig. 4. In Figs. 4a,b the power spectrum as a function of *ω* and *ω*_*D*_ is presented for *d* = 10nm and *v* = 0. The effects of finite drift are shown in Figs. 4c,d, where the normalized contrast $\Delta S=1-\frac{{S}_{v}(\omega )}{{S}_{v=0}(\omega )}$ is plotted as function of the same parameters. Since the most likely experiment would be to measure first the spectrum without drift and then to perform a second measurement with finite drift and compare the two, the normalized contrast is the most relevant quantity.

In the intermediate frequency regime, for *ω* ≈ *ω*_*D*_, the uncertainty is

3 $$\Delta {\tilde{v}}=\frac{1}{\sqrt{T}}\frac{1}{\sqrt{{\tau }_{D}}{\gamma }_{e}{B}_{RMS}}.$$

This constitutes an improvement by a large factor of  ${\tilde{v}}^{-1}=\frac{{\omega }_{D}}{{\omega }_{v}}$ in comparison to Eq. 2. Substituting the parameters for water we estimate $\Delta {v}_{s}=10\frac{{\rm{mm}}}{{\rm{s}}}\sqrt{\frac{{\rm{s}}}{T}}$ for 15nm deep NV, which is the optimal depth as the power spectrum at this depth equals ${T}_{2}^{-1}$. For an ensemble of NVs we achieve the enhancement $\Delta {v}_{ens}=1\frac{{\rm{mm}}}{{\rm{s}}}\sqrt{\frac{{\rm{s}}}{T}}\sqrt{\frac{{(\mu {\rm{m}})}^{2}}{A}}$. To appreciate this scaling, consider that for water the dimensionless parameter ${\tilde{v}}=1$ corresponds to a drift velocity of $v=150\,\frac{{\rm{mm}}}{{\rm{s}}}.$

In the low frequency region the decay rate is equal to

4 $$\widetilde{S}=1-\sqrt{\tilde{v}}$$

and the uncertainty is therefore,

5 $$\Delta {\tilde{v}}=\frac{2}{{\gamma }_{e}{B}_{RMS}\sqrt{{\tau }_{D}}}\sqrt{\frac{\tilde{v}}{T}}$$

which is a substantial improvement by a factor of $\sqrt{\tilde{v}}$ compared to the intermediate range (Eq. 3). This regime is applicable whenever ${T}_{2}\gg \frac{1}{{\omega }_{v}}$, which is attainable for low viscosity fluids as this time scale is around 10*μ*s in that case. The sensitivity in this regime, $\frac{\Delta v}{v}=\frac{2}{{\gamma }_{e}{B}_{RMS}{\tau }_{D}}\sqrt{\frac{d}{Tv}}$, is a decreasing function of *v* as expected. Using the same parameters for water, and *d* = 15nm the sensitivity is estimated by $\frac{\Delta {v}_{ens}}{v}=0.15\frac{\sqrt{{\rm{s}}}}{\sqrt{T}}\sqrt{\frac{{(\mu m)}^{2}}{A}}$. This result represents an improvement by more then three orders of magnitude in fractional uncertainty over the Lorentzian model.

### Molecular dynamics

In order to verify our analytical results we performed a molecular dynamics simulation. The simulated system consisted of N interacting particles confined to a box and the NV was placed a distance *d* below the simulation box at the center of the x-y projection of the box.

**Figure 5:**
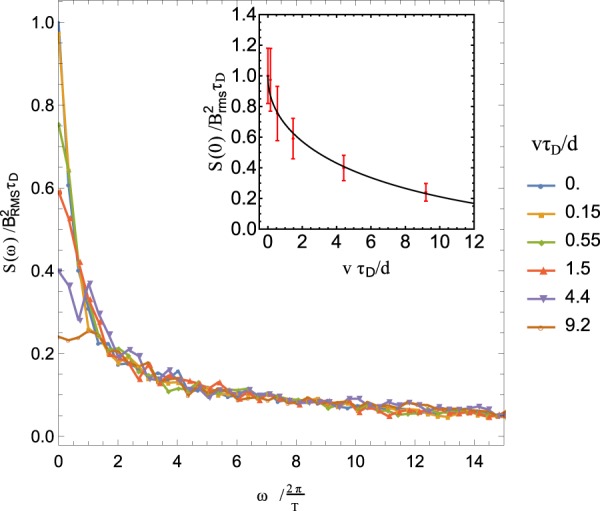
Power spectra *S*(*ω*) obtained using the LJ fluid model for six different values of drift velocity v. The average $S(\omega )=\left\langle | {\mathscr{F}}[{B}_{z}(t)]{| }^{2}\right\rangle$ was taken over 130 simulation runs. The inset shows the sensitivity of the power spectrum at zero frequency on the drift velocity. The data were numerically fitted to a function $1+b\sqrt{v}+cv$, with *b* = −0.42 and *c* = 0.053 which yielded *R*^2^ = 0.9926. The error bars indicate the 95% confidence intervals. Each point corresponds to the averaging of 130 runs.

 Figure 5 shows the power spectra of the magnetic field. The value of the power spectrum at *ω* = 0 for different velocities, as shown in the inset of the figure, was fitted to the function $1+b\sqrt{v}+cv$, with *b* = −0.42 and *c* = 0.053 which yielded *R*^2^ = 0.9926, validating Eq. 4 and the sensitivity estimation (Eq. 5). The value of *b* obtained by the simulation does not match Eq. 4 where *b* = −1 because the latter was derived using approximate scaling arguments (see SI). A full description of the simulation parameters is given at the Methods section.

### Comparison to fluorescent based velocimetry

Current methods for velocity measurement in microfludic channels are based mostly on fluorescent beads or dyes. Typically, fluorescent molecules are injected into the channel and their propagation is tracked using a confocal microscope^60–65^. Alternatively, the velocity can be determined by light scattering from a periodic array^66^. Fluorescent molecules can also be used to measure the diffusion coefficient of these molecules within a liquid^64^. These methods lack in performance and only achieve an accuracy of about 5% for the mean channel velocity due to various technical issues which include laser beam focusing and the temporal resolution of the camera. Though progress in technology may help overcome these limitations in the future, these methods suffer from fundamental issues as well. The fluorescent molecules are often large, and in a small channel they can affect the flow profile. In addition, the flow trajectories of these molecules generally pass through the middle of the tube because of the velocity gradient, which supplies little information about the flow profile near the surface. Finally, current methods can only be used to measure the diffusion coefficient of the fluorescent molecule within the liquid, whereas the interesting parameters are usually the self-diffusion and rotational diffusion coefficients of the liquid. Our proposed setup solves these problems since it is non-intrusive and sensitive to surface effects.

To verify our claim that our method outperforms the fluoresce based techniques we note that they all suffer from a limitation on the molecule size. The relative error that is caused by the Brownian motion can be estimated by^61^

6 $$\varepsilon =\frac{{\rm{Diffusion}}\ {\rm{distance}}}{{\rm{Drift}}\ {\rm{distance}}}=\frac{1}{v}\sqrt{\frac{2D}{T}},$$

where *T* is the experiment time. Since the diffusion is an unbiased noise, this error can be reduced by averaging over *N* different particles

7 $$\varepsilon =\frac{1}{v}\sqrt{\frac{2D}{NT}},$$

where *N*, is still limited by systematic errors. The distance between the molecules, provides these system a characteristic length, *d*_*c*_, which is bounded by the molecule’s size. This enables us to write (7) in a similar way to our previous assessments,

8 $$\varepsilon =\frac{1}{v/{d}_{c}}\sqrt{\frac{2D}{{d}_{c}^{2}NT}}=\frac{1}{{\omega }_{v}}\sqrt{\frac{{\omega }_{D}}{NT}}=\frac{1}{\tilde{v}}\sqrt{\frac{1}{N{\omega }_{D}T}}.$$

We note that the relative error, Eq. 8, has the same scaling in ${\tilde{v}}$ as the one in the Lorentzian model given by Eq. 2. Therefore, these methods will not be able to estimate velocities for which ${\tilde{v}} < 1$. The lower limit will be given by ${v}_{c}=\frac{D}{{d}_{c}}$. This is problematic as *d*_*c*_ should be small enough, for the molecules not to change the flow profile in the channel, but it has to be large enough to eliminate the effect of diffusion. This poses a fundamental problem, which cannot be overcome. Our method provides an improved scaling in ${\tilde{v}}$ even when it is small.

## Conclusion

Here we proposed a new setup to study flow properties in micro- and nano - fluidic structures which meets a crucial need in the fast growing field of microfluidics. The proposed setup is quantum inspired and is based on quantum sensing via color centers in solids. The proposal relies only on statistical polarization and therefore can be easily implemented. Our setup outperforms current velocimetry methods as it is sensitive to surface effects and it is effective even when diffusion dominates the dynamics.

## Methods

### The correlation function and power spectrum of diffusing particles

The dipole-dipole interaction can be decomposed into six parts associated with angular momentum exchange between the NV and a nucleus. Each of these terms rotates at a different frequency when the NV is driven by an external field and therefore contributes to a different part of the power spectrum.

This leads to the definition of the correlation functions,

9 $${G}^{\left({m}_{1},{m}_{2}\right)}(t)=\int \int {f}^{\left({m}_{1}\right)* }\left(\bar{r}\right){f}^{\left({m}_{2}\right)}\left({\bar{r}}_{0}\right)P\left(\bar{r},t| {\bar{r}}_{0},0\right)P\left({\bar{r}}_{0}\right){d}^{3}r{d}^{3}{r}_{0},$$

where where ${f}^{(m)}(\bar{r})\propto \frac{1}{{r}^{3}}{Y}_{2}^{(m)}\left(\Omega \right)$ are the spatial dependencies of the magnetic field given by the Spherical Harmonics, $P({\bar{r}}_{0})$ is the initial spatial distribution of the nuclear spin ensemble, assumed throughout this work to be uniform, and $P\left(\bar{r},t| {\bar{r}}_{0},0\right)$ is the propagator from the point ${\bar{r}}_{0}$ to $\bar{r}$ given a time difference *t*. The dimensionless correlation functions ${G}^{({m}_{1},{m}_{2})}$ are equal to the correlation functions of the magnetic field up to a multiplicative factor of $J\equiv {\left(\frac{{\mu }_{0}{\gamma }_{N}\hslash }{4\pi }\right)}^{2}$. The first step in calculating these correlation functions is to find the appropriate propagator. Assuming, the ensemble is freely diffusing in the half space above the diamond surface, the propagator *P* will be the solution to the diffusion equation in the half space with the initial condition $P\left(\bar{r},t=0| {\bar{r}}_{0},0\right)=\delta \left(\bar{r}-{\bar{r}}_{0}\right)$. Solving for the whole space and using the method of images we find

10 $$P(\bar{r},t| {\bar{r}}_{0},0)={(4\pi Dt)}^{-3/2}{e}^{-\frac{{\left(x-{x}_{0}\right)}^{2}}{4Dt}}{e}^{-\frac{{\left(y-{y}_{0}\right)}^{2}}{4Dt}}\left({e}^{-\frac{{\left(z-{z}_{0}\right)}^{2}}{4Dt}}+{e}^{-\frac{{\left(z+{z}_{0}-2d\right)}^{2}}{4Dt}}\right).$$

If the NV is not tilted, meaning, it’s magnetization axis coincides with the normal to the diamond surface, the only correlation functions that do not equal zero are

11 $${G}^{(m)}\equiv {G}^{(m,m)}.$$

Calculating Eq.11 by performing the integrals in Eq. 9 explicitly we find that

12 $${G}^{(0)}=4{G}^{(1)}=16{G}^{(2)},$$

and

13 $$\begin{array}{cc}{G}^{(0)} & =\,n{\pi }^{1/2}[\frac{1}{{(Dt)}^{3/2}}-\frac{3}{2{d}^{2}\sqrt{Dt}}+\frac{3\sqrt{Dt}}{{d}^{4}}-\frac{3Dt\sqrt{\pi }}{2{d}^{5}}\\  & \,+\sqrt{\pi }Erfc\left(\frac{d}{\sqrt{Dt}}\right){e}^{\frac{{d}^{2}}{Dt}}\left(-,\frac{d}{{(Dt)}^{2}},+,\frac{1}{d(Dt)},-,\frac{7}{4{d}^{3}},+,\frac{3Dt}{2{d}^{5}}\right)+\frac{\sqrt{\pi }}{4{d}^{3}}].\end{array}$$

The asymptotic behavior of  Eq. 13 is given by

14 $${G}^{(0)}\approx \{\begin{array}{cc}\frac{n\pi }{4{d}^{3}} & t\ll {\tau }_{D}\\ \frac{8n}{15}\frac{{\pi }^{1/2}}{{(Dt)}^{3/2}} & t\gg {\tau }_{D}\end{array}=\frac{{B}_{RMS}^{2}}{{J}^{2}}\{\begin{array}{cc}1 & t\ll {\tau }_{D}\\ \frac{32}{15\sqrt{\pi }}{\left(\frac{{\tau }_{D}}{t}\right)}^{3/2} & t\gg {\tau }_{D}\end{array}.$$

The instantaneous correlation defines the *B*_*RMS*_, but the long time limit, often assumed to decay exponentially, is actually given by a power law.

The power spectrum around *ω* = 0 will be give by the Fourier transform of Eq. 13,

15 $$\begin{array}{ccc}S(\omega ) & = & n{\gamma }_{e}^{2}{J}^{2}\frac{\pi }{\sqrt{2}}\{\frac{8}{5}\frac{{|\omega |}^{1/2}}{{D}^{3/2}}Re\left({}_{1},{F}_{4},(1;\frac{3}{4},\frac{5}{4},\frac{7}{4},\frac{9}{4};-\frac{{d}^{4}{\omega }^{2}}{16{D}^{2}})\right)\\  &  & -\frac{56}{225}\frac{{|\omega |}^{3/2}{d}^{2}}{{D}^{5/2}}Re\left({}_{1},{F}_{4},(1;\frac{7}{4},\frac{7}{4},\frac{9}{4},\frac{9}{4};-\frac{{d}^{4}{\omega }^{2}}{16{D}^{2}})\right)\\  &  & -\frac{224}{225}\frac{{|\omega |}^{3/2}{d}^{2}}{{D}^{5/2}}Re\left({}_{1},{F}_{4},(2;\frac{7}{4},\frac{7}{4},\frac{9}{4},\frac{9}{4};-\frac{{d}^{4}{\omega }^{2}}{16{D}^{2}})\right)\\  &  & +\frac{32}{105}\frac{{|\omega |}^{3/2}{d}^{2}}{{D}^{5/2}}Re\left({}_{1},{F}_{4},(1;\frac{5}{4},\frac{7}{4},\frac{9}{4},\frac{11}{4};-\frac{{d}^{4}{\omega }^{2}}{16{D}^{2}})\right)\\  &  & -\frac{64}{225}\frac{{|\omega |}^{5/2}{d}^{4}}{{D}^{7/2}}Re\left({}_{1},{F}_{4},(1;\frac{7}{4},\frac{7}{4},\frac{9}{4},\frac{9}{4};-\frac{{d}^{4}{\omega }^{2}}{16{D}^{2}})\right)\\  &  & +\frac{32}{225}\frac{{|\omega |}^{5/2}{d}^{4}}{{D}^{7/2}}Re\left({}_{1},{F}_{4},(1;\frac{7}{4},\frac{9}{4},\frac{9}{4},\frac{11}{4};-\frac{{d}^{4}{\omega }^{2}}{16{D}^{2}})\right)\\  &  & +\frac{128}{1575}\frac{{|\omega |}^{7/2}{d}^{6}}{{D}^{9/2}}Re\left({}_{1},{F}_{4},(1;\frac{7}{4},\frac{9}{4},\frac{9}{4},\frac{11}{4};-\frac{{d}^{4}{\omega }^{2}}{16{D}^{2}})\right)\\  &  & -\frac{256}{11025}\frac{{|\omega |}^{7/2}{d}^{6}}{{D}^{9/2}}Re\left({}_{1},{F}_{4},(1;\frac{9}{4},\frac{9}{4},\frac{11}{4},\frac{11}{4};-\frac{{d}^{4}{\omega }^{2}}{16{D}^{2}})\right)\\  &  & -\frac{512}{99225}\frac{{|\omega |}^{9/2}{d}^{8}}{{D}^{11/2}}Re\left({}_{1},{F}_{4},(1;\frac{9}{4},\frac{11}{4},\frac{11}{4},\frac{13}{4};-\frac{{d}^{4}{\omega }^{2}}{16{D}^{2}})\right)\\  &  & +\frac{3D}{\sqrt{2}{\omega }^{2}{d}^{5}}-\frac{8}{3}\frac{{|\omega |}^{1/2}}{{D}^{3/2}}-\frac{8}{9}\frac{{|\omega |}^{3/2}{d}^{2}}{{D}^{5/2}}+\frac{32}{45}\frac{{|\omega |}^{5/2}{d}^{4}}{{D}^{7/2}}\\  &  & -\frac{3\sqrt{2}}{|\omega |{d}^{3}}\left[\frac{\pi }{2},{{\rm{b}}{\rm{e}}{\rm{r}}}_{2},(2\sqrt{\frac{|\omega |}{D}}d),+,{kei}_{2},(2\sqrt{\frac{|\omega |}{D}}d)\right]\\  &  & +\frac{2\sqrt{2}}{Dd}\left[-,\frac{\pi }{2},{\rm{b}}{\rm{e}}{\rm{i}},(2\sqrt{\frac{|\omega |}{D}}d),+,k,e,r,(2\sqrt{\frac{|\omega |}{D}}d)\right]\\  &  & +\frac{7}{2}\frac{1}{{|\omega |}^{1/2}{D}^{1/2}{d}^{2}}[{ker}_{1}\left(2,\sqrt{\frac{|\omega |}{D}},d\right)-{kei}_{1}\left(2,\sqrt{\frac{|\omega |}{D}},d\right)-\frac{\pi }{2}{{\rm{b}}{\rm{e}}{\rm{i}}}_{1}\left(2,\sqrt{\frac{|\omega |}{D}},d\right)\\  &  & -\frac{\pi }{2}{{\rm{b}}{\rm{e}}{\rm{r}}}_{1}\left(2,\sqrt{\frac{|\omega |}{D}},d\right)]\\  &  & -2\frac{{|\omega |}^{1/2}}{{D}^{3/2}}[\frac{\pi }{2}{{\rm{b}}{\rm{e}}{\rm{r}}}_{1}\left(2,\sqrt{\frac{|\omega |}{D}},d\right)-\frac{\pi }{2}{{\rm{b}}{\rm{e}}{\rm{i}}}_{1}\left(2,\sqrt{\frac{|\omega |}{D}},d\right)+{kei}_{1}\left(2,\sqrt{\frac{|\omega |}{D}},d\right)\\  &  & +{ker}_{1}\left(2,\sqrt{\frac{|\omega |}{D}},d\right)]\},\end{array}$$

where ${}_{p}{F}_{q}\left(\left\{{a}_{1},\cdots \ ,{a}_{p}\right\};\left\{{b}_{1},\cdots \ ,{b}_{q}\right\};z\right)$ is the generalized hypergeometric function and ber_*n*_(*x*)/bei_*n*_(*x*)/*ker*_*n*_(*x*)/*kei*_*n*_(*x*) are the Kelvin functions of order *n*. The asymptotic behavior of  Eq. 15 when $\left|\omega \right|\ll {\omega }_{D}$ is

16 $$S(\omega )\approx {\gamma }_{e}^{2}{B}_{RMS}^{2}{\tau }_{D}\left(\frac{3}{2}-\frac{32\sqrt{2}}{15}\sqrt{\frac{\left|\omega \right|}{{\omega }_{D}}}+\frac{5\pi }{4}\frac{\left|\omega \right|}{{\omega }_{D}}\right).$$

Note that the leading term in the expansion (Eq. 16) in the parameter $\frac{\left|\omega \right|}{{\omega }_{D}}$ is the Fourier transform of the long time limit (Eq. 14) as expected. The large frequency regime is hard to derive from the general expression (Eq. 15) as the special functions strongly diverge at large arguments. We deduce the this behavior by other arguments (see SI).

17 $$S\left(\omega \right)\propto {\gamma }_{e}^{2}{B}_{RMS}^{2}{\tau }_{D}{\left(\frac{{\omega }_{D}}{\omega }\right)}^{2},$$

where the proportion constant cannot be determined by our estimation. Since Eq. 17 is consistent with the Lorentzian model, measuring the deviation from this model requires an accurate measurement of the power spectrum at zero frequency or at the Larmor frequency by Eq. 12. A heuristic sketch of the power spectrum is shown in Fig. 3. Detailed derivation of the results of this section and the following are found in the SI.

### Corrections to the power spectrum at low drift

The calculation of the Fourier transform of the temporal correlation function with finite drift is difficult. Hence, we chose to estimate the behavior of the power spectrum directly by taking the Fourier transform of Eq. 9,

18 $${S}^{({m}_{1},{m}_{2})}(\omega )=\int \int {f}^{\left({m}_{1}\right)* }\left(\bar{r}\right){f}^{\left({m}_{2}\right)}\left({\bar{r}}_{0}\right)P\left(\bar{r},\omega | {\bar{r}}_{0},0\right)P\left({\bar{r}}_{0}\right){d}^{3}r{d}^{3}{r}_{0},$$

where $P\left(\bar{r},\omega | {\bar{r}}_{0},0\right)$ is the diffusion propagator in the Fourier domain. Assuming the nuclei flow in a constant velocity $\bar{v}$, the propagator is given by

19 $$P(\bar{r}| {\bar{r}}_{0},\omega )=\frac{1}{4\pi D\left|\bar{r}-{\bar{r}}_{0}\right|}{e}^{i{k}_{1}\cdot \left|\bar{r}-{\bar{r}}_{0}\right|}{e}^{\frac{\bar{v}\cdot \left(\bar{r}-{\bar{r}}_{0}\right)}{2D}}.$$

with

20 $$\phi =\pi -ta{n}^{-1}\left(\frac{4D\omega }{{v}^{2}}\right),R=\sqrt{{\left(\frac{v}{2D}\right)}^{4}+{\left(\frac{\omega }{D}\right)}^{2}},\ {k}_{1}=\sqrt{R}{e}^{i\phi /2}.$$

The propagator, Eq. 19, can be expanded into a series in the low drift limit $\frac{{\omega }_{v}^{2}}{\omega {\omega }_{D}}\ll 1$. Taking advantage of the non-Lorentzian spectrum and requiring the series expansion to converge yields further restrictions - *ω*_*v*_ ≪ *ω* ≪ *ω*_*D*_. Assuming the direction of the velocity is restricted to the *x* − *y* plane, in this regime, the expansion of the spectrum of an untilted NV will be

21 $${S}_{v}(\omega )\approx {S}_{0}(\omega )+O({v}^{2}).$$

The linear contribution in the velocity vanishes, because of the polar symmetry of the Spherical Harmonics. Since the velocity scaling is similar to the Lorentzian model, no advantage is gained in the sensitivity when the NV is untilted.

However, when the NV is tilted the polar symmetry is broken and the expansion reads

22 $${S}_{v}\left(\omega \right)\approx {S}_{0}\left(\omega \right)\left(1-C\frac{{\omega }_{v}}{{\omega }_{D}}\sqrt{\frac{{\omega }_{D}}{\omega }}\right),$$

where *C* is some constant that we were unable to evaluate. Extending Eq. 22 to *ω* ~ *ω*_*v*_ by taking *ω* = *ω*_*v*_ results in

23 $${S}_{v}\left(\omega \right)\approx {S}_{0}\left(\omega \right)\left(1-C\sqrt{\frac{{\omega }_{v}}{{\omega }_{D}}}\right).$$

Analytic calculation of the power spectrum in another geometry and the molecular dynamics simulations support the scaling found in Eq. 23 (see SI). A heuristic sketch of the power spectrum in the low drift limit is shown in Fig. 3b.

### Molecular dynamics

**Figure 6:**
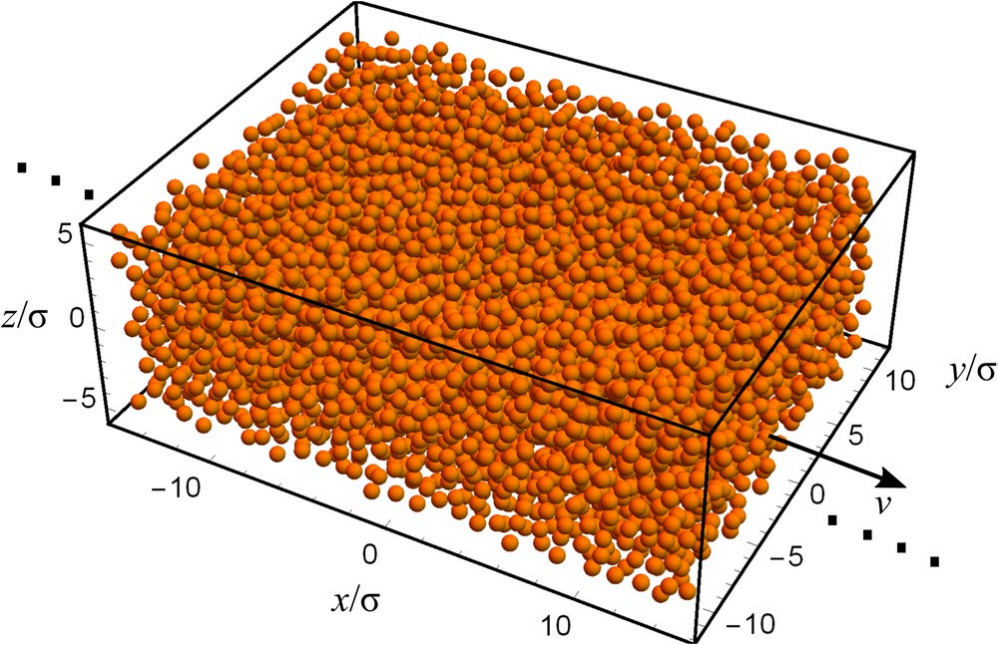
The dipole bath is simulated using LJ fluid model. The thermal motion results in fluid self-diffusion. A drift velocity *v* is added to every particle at the start of the simulation. The dimensions of the simulation box were *L*_*x*_ = 163.08*σ*, *L*_*y*_ = 22.68*σ* and *L*_*z*_ = 10.8*σ*. The number of particles was 31710 giving the density of *ρ* = 0.79*σ*^−3^.

 Figure 6 illustrates the simulation setup. The system consists of N dipolar particles confined to a simulation box of volume *L*_*x*_*L*_*y*_*L*_*z*_. Particles are interacting via the Lennard Jones (LJ) potential $4\varepsilon \left[{\left(\frac{\sigma }{r}\right)}^{12}-{\left(\frac{\sigma }{r}\right)}^{6}\right]$ with an interaction cut-off distance of *r*_*c*_ = 2.5*σ*. The system is initialized into a thermal state at temperature *T* by applying a Langevin thermostat and each particle is assigned a random spin value *I*_*z*_ ∈ {−1, 1}. After thermalization, the average particle velocity is subtracted from each particle, e.g. ${\bar{v}}_{j}\leftarrow {\bar{v}}_{j}- < \,\bar{v} >$. The initialization concludes with the addition of the drift velocity *v* in the x-direction to each particle *v*_*xj*_ ← *v*_*xj*_ + *v*. The simulation dynamics is deterministic, following Newton’s laws, and integrated using the Velocity-Verlet method with step size $\Delta t=0.00125\sqrt{\varepsilon /(m{\sigma }^{2})}$. During the simulation the *z* component of the magnetic field induced by the particles at the position of the NV center is computed using the equation

24 $$B(t)={\sum }_{i=1}^{N}\frac{1}{{r}_{i}^{3}(t)}\left[3{\cos }^{2}({\theta }_{i}(t))-1\right]{I}_{z}^{i},$$

where *r*_*i*_ is the distance between particle *i* and the NV center, and *θ*_*i*_ is the angle between *r*_*i*_ and the NV’s quantization axis. The NV is placed a distance *d* below the simulation box in the center of the xy plane. Specular reflections are applied at the boundaries that are perpendicular to the z-axis and periodic boundary conditions are used in x and y directions. To emulate particles coming into and leaving the simulation box, we flip the spin of particles that reach one of the periodic boundary walls with probability 1/2.

Figure 5 shows the power spectra computed using the LJ fluid model for *d* = *σ* and *L*_*x*_ = 163.08*σ*. The diffusion time scale *τ*_*D*_ is computed from the relation $S(\omega =0;v=0)={B}_{{\rm{R}}{\rm{M}}{\rm{S}}}^{2}{\tau }_{D}$. The integration was carried out for 3.2 × 10^6^ time steps, which resulted in total integration time of 18.7*τ*_*D*_. The value of the power spectrum at *ω* = 0 for different velocities, shown in the inset of the figure, was fitted using least-squares algorithm.

## Supplementary information

Supplementary Information.
